# SCOBY Cellulose-Based Materials Hydrophobized Using Stearic Acid and Apple Powder

**DOI:** 10.3390/ijms252413746

**Published:** 2024-12-23

**Authors:** Malgorzata Anita Bryszewska, Daniel Gutierez Pareja, Lukasz Kaczmarek, Anna Sobczyk-Guzenda, Malgorzata Piotrowska, Damian Batory

**Affiliations:** 1 Institute of Natural Products and Cosmetics, Faculty of Biotechnology and Food Sciences, Lodz University of Technology, 90-537 Lodz, Poland; 2 Faculty of Pharmacy, Universitario de Cartuja, University of Granada, 18011 Granada, Spain; dani11playgp@gmail.com; 3 Faculty of Mechanical Engineering, Institute of Materials Science and Engineering, Lodz University of Technology, 90-924 Lodz, Poland; lukasz.kaczmarek@p.lodz.pl (L.K.); anna.sobczyk-guzenda@p.lodz.pl (A.S.-G.); 4 Faculty of Biotechnology and Food Sciences, Institute of Fermentation Technology and Microbiology, 90-530 Lodz, Poland; malgorzata.piotrowska@p.lodz.pl; 5 Department of Vehicles and Fundamentals of Machine Design, Lodz University of Technology, 90-924 Lodz, Poland; damian.batory@p.lodz.pl

**Keywords:** bacterial cellulose, SCOBY, apple powder, hydrophobization, stearic acid, biodegradable materials

## Abstract

Bacterial cellulose (BC) is a subject of interest for researchers due to its advantageous characteristics, including a straightforward manufacturing process, biocompatibility, and extensive modification potential. The hydrophilic nature of the material is beneficial in some applications, yet a limiting factor in others. This study aimed to develop BC-based materials with goFogureod moisture resistance. The modification of bacterial cellulose (BC) using apple powder, stearic acid, or a combination of these modifiers resulted in the formation of a range of materials, some of which had their surfaces additionally functionalised by coating with a mixture of apple powder and stearic acid (HSt). The nature and type of changes were confirmed by FTIR and theoretical analysis, which was conducted by modelling the interaction between cellulose and homogalacturonan or rhamnogalacturonan using SCIGRESS v.FJ 2.7 software. Changes in hydrogen bonding resulting in a weakening of the interactions between cellulose and water in the presence of pectin were demonstrated by both empirical data and modelling. The effectiveness of BC functionalisation was confirmed by material wettability. The water contact angle changed from 38° for the unmodified material to 125° for the material obtained by modification of the bacterial cellulose with glycerol followed by modification with a mixture of HSt at a concentration of 10% and AP at a concentration of 60%. The modifications produced a material with a robust hydrophobic surface. The results suggest that the surface roughness may not be the primary factor influencing the hydrophilicity or hydrophobicity of these materials but that it is more likely to be related to the interactions of components. None of the tested materials demonstrated antimicrobial activity against *Escherichia coli*, *Bacillus subtilis*, *Staphylococcus aureus*, *Aspergillus niger*, or *Candida albicans*.

## 1. Introduction

The currently outlined development directions involve the utilisation of waste materials in the creation of novel products. This is in response to the identified need for the development of biodegradable, non-petroleum-based, carbon-neutral products that originate from renewable and sustainable sources [1]. Among those type of sources, cellulose is of particular interest due to its ability to fulfil a considerable proportion of the aforementioned requirements. The natural occurrence of cellulose is not confined to a single origin. The most abundant and common used cellulose is that derived from plant sources, such as that obtained from wood, as well as from cotton and bamboo. Nevertheless, the capacity to produce this polysaccharide is not exclusive to the plant kingdom. A number of bacterial species are also capable of synthesizing cellulose as an extracellular biopolymer. The most well known of these are bacteria belonging to the *Acetobacter*, *Achromobacter*, *Komagataeibacter*, *Agrobacterium*, *Bacillus*, *Azotobacter*, *Sarcinia*, *Lactobacillus*, and *Gluconacetobacter* genera or microbial consortia such as those that form SCOBY (Symbiotic Colony of Bacteria and Yeasts) [2,3]. This symbiotic colony is characterised by biological stability, resistance to contamination, and its applicable by-products: a probiotic brew and bacterial cellulose [3]. The biofilm of BC is a naturally occurring byproduct of the juice fermentation or kombucha beverage fermentation process, manifesting as a pellicle at the air–liquid interface. Sustainable and green economy requirements are perfectly met by cellulose produced by SCOBY, since this method of production does not require the availability of a specific bacterial strain(s); it is accessible, even under domestic conditions; and the fermentation process is eco-friendly and cost-effective. All these advantages are in line with the principles of green chemistry processes that reduce or eliminate the use or generation of hazardous substances, making the process safe for the human body [1]. In regard to its composition and structure, the bacterial biopolymer is analogous to plant cellulose. BC has the same chemical structure, (C_6_H_10_O_5_)n, but with a nano-sized polymer network structure, high purity (96–98%), high crystallinity (84–91%), and a high degree of polymerization (ranging from 2000 to 6000) [2,4,5]. In contrast to plant cellulose, BC does not contain lignin or hemicellulose. Consequently, the preparation process is relatively straightforward and can be accomplished with minimal effort through the use of mild treatment [6]. Additionally, BC possesses exceptional physicochemical properties, such as high crystallinity, high specific surface area, high elasticity, relatively high mechanical strength in the wet state, hydrophilicity, and excellent biocompatibility [7,8]. These distinctive characteristics render BC an appealing proposition from a technological, ecological, and financial standpoint. The substantial potential of this biopolymer has been successfully actualized in a multitude of applications [9,10,11]. Its properties, such as biocompatibility, flexibility, high water retention, and good cell adhesion, have led to the current commercialization of BC as a wound dressing material for tissue regeneration [12,13,14,15]. The broadening of its range of applications requires modification and the incorporation of other molecules. The most straightforward and efficacious method and, therefore, the one most commonly employed, entails the introduction of drugs into BC membranes through immersion in a drug solution, which is typically followed by lyophilization [16,17]. The drugs most frequently incorporated into BC membranes by this by this method are anti-inflammatory agents such as ibuprofen and diclofenac, as well as antimicrobial drugs [16,18,19]. The mechanical properties of BC can be enhanced through the application of physical methods, such as rotating magnetic fields and ultrasound treatments, which have enabled the production of cellulose with enhanced water absorption and density [20] or stability [21]. Chemical modifications of BC are being developed extensively, with the latest trends including the oxidation of hydroxyl groups of cellulose (e.g., using 2,2,6,6-tetramethyl-1-piperidinyloxyl [22], chemical grafting of aminoalkyl groups [23], and esterification [24]. Additionally, there has been significant progress in the design of BC composite formulations. These can be obtained by combining BC with other polymers through in situ procedures (e.g., hyaluronic acid) [25] or by ex situ synthesis, as exemplified by chitosan [26]. Such methods were reviewed by Blanco Parte et al. [11].

A significant area of research within the field of cellulose modifications is dedicated to the development of materials with enhanced hydrophobic characteristics. By nature, BC is highly hydrophilic due to its porous structure (porosity of 83–86% [2]; fibrous polymer with a large surface area) and chemical composition (three highly polar hydroxyl groups in each glucose residue). To improve moisture resistance and barrier properties and to maintain these properties for expanded applications such as textiles or packaging, BC needs to be surface-functionalised. This objective can be attained with reasonable ease either by incorporating hydrophobic synthetic substances into the cellulose structure or by coating it with such substances. However, each time this type of substance is introduced to the biopolymer, there is a possibility of the alteration of its toxicity or bio-utilisation capacity. Although more challenging, significant increases in the hydrophobicity and elasticity of bacterial cellulose have been achieved by incorporating natural and biodegradable components into the polymer, such as beeswax [27] or montmorillonite [28]. Beeswax is a natural polymer and, as a consequence, is deemed a biodegradable material, with its decomposition largely facilitated by waxworms in the natural environment [29]. Montmorillonite is a naturally occurring aluminosilicate mineral that is considered to have an environmentally safe profile. When incorporated into bio-composites, it has been demonstrated to enhance their mechanical properties [28].

A second highly desirable and naturally absent property of BC-based materials is antimicrobial activity. Microbicidal cellulose can be produced by introducing the functional agent onto the surface by treatment methods such as spray coating followed by fixation of the agent, impregnation, or the introduction of the agent directly into the polymer matrix. Examples of effective modifications already described include enrichments of BC with nanosilver [28], benzalkonium chloride [28], and silver sulphadiazine [30].

In the previously presented work, the possibility of modifying the properties of BC by introducing apple powder and glycerol into the biopolymer structure was investigated [31]. A combination of pragmatic, economic, and structural considerations led to the selection of AP as a modifier of the properties of BC. AP represents a waste material generated by the fruit industry that is both accessible and cost-effective. The similarities in the chemical composition of pectin and cellulose bring about the possibility of chemical bonding between the biopolymers. A durable so-called apple leather material was obtained with satisfying mechanical properties. This work presents further modifications. Maintaining the key assumption that the developed material should be entirely natural, stearic acid was chosen as a potential hydrophobization component. This decision was based on observations in nature, where the wax coating of fruits forms a protective barrier against water loss and microbial attack.

## 2. Results and Discussion

### 2.1. Visual and Sensory Characteristics of Materials

**Figure 1:**
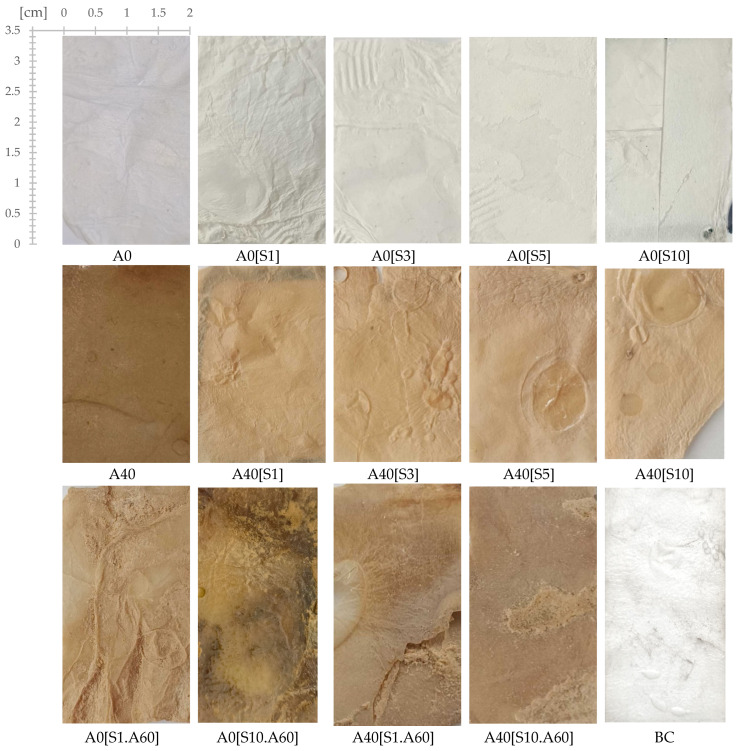
Images of materials. Samples codes: BC—bacterial cellulose (biopolymer after fermentation process, pre-treatment); A0—bacterial cellulose modified with glycerol; A0[S1], A0[S3], A0[S5], and A0[S10]—bacterial cellulose modified with glycerol followed by HSt at a concentration indicated by the bracketed numbers; A40—bacterial cellulose modified with a mixture of glycerol and AP at a concentration indicated by the number; A40[S1], A40[S3], A40[S5], and A40[S10]—bacterial cellulose modified with a mixture of glycerol and AP followed by modification with HSt at a concentration indicated by the bracketed numbers; A0[S1.A60] and A0[S10.A60]—bacterial cellulose modified with glycerol followed by modification with a blend of HSt at a concentration of 1% and 10%, respectively (indicated by the respective numbers), and AP at a concentration of 60%; A40[S1.A60] and A40[S10.A60]—bacterial cellulose modified with a mixture of glycerol and AP followed by modification with a mixture of HSt at a concentration of 1% and 10%, respectively (indicated by the respective numbers), and AP at a concentration of 60%.

The digital images of the BC and its modifications are presented in Figure 1. The resulting biopolymer displays a yellowish colouration, which is attributable to the presence of polyphenols in the culture medium and a distinctive aroma of organic acids produced by the symbiotic culture during the tea fermentation process [3]. Pure bacterial cellulose (BC) is obtained by removing the residue of the culture medium during pre-treatment by washing and bleaching. This treatment produces a white, slightly transparent material with a parchment-like appearance.

The modification of BC with glycerol (A0) as a plasticiser yields a white, non-transparent, and plastic material. Sample A0 has a smooth texture and a slightly oily feel. The addition of glycerol increased the flexibility of the product compared to plain kombucha biofilm.

Subsequent modification with HSt in concentrations ranging from 1 to 10% produced opaque white materials (A0[S1], A0[S3], A0[S5], and A0[S10]), which all provided a dry, nonslip feel upon touch. Moreover, all of these materials were prone to fracturing and crumbling, with the greatest degree of the latter being observed at the lowest HSt concentrations.

The modification of bacterial cellulose with apple powder (A40) resulted in material with a brown colour similar to that of dried apple pulp, with small areas of discolouration and a persistent apple aroma. The texture of the material was also improved, providing a pleasant and non-greasy sensation, as observed for A0, while still maintaining high pliability. Subsequent modification of A40 with a layer of stearic acid applied in varying concentrations resulted in A40[S1], A40[S3], A40[S5], and A40[S10], with the number in brackets indicating the concentration of HSt in the modifying solution. HSt modification is reflected by the colour change of the samples. As the concentration of HSt increases, the colour becomes slightly lighter. Modified samples gave the impression of being dry and rough. To illustrate this sensation, it could be compared to touching dried autumn leaves. Starting with a sample containing 3% HSt, the perception of dryness is diminished, and the perception of slippage is enhanced as the concentration of HSt rises, whereas for samples containing between 5% and 10% HSt, the difference is negligible. Furthermore, that group of materials demonstrated poor flexibility and was prone to cracking, a tendency that was heightened with higher concentrations of HSt. Interestingly, the material’s appearance underwent a significant transformation upon the application of a water droplet. The treated area exhibited a lighter and marginally translucent texture, with the changes remaining, even after drying.

Materials created by modification with glycerol followed by treatment with a blend of HSt at a concentration of 1% or 10% (indicated by the respective numbers in the sample code) and AP at a concentration of 60% (A0[S1.A60] and A0[S10.A60]) exhibited an attractive brown hue, together with superior elasticity and fracture resilience when compared to the other materials. Lastly, materials obtained by modification of bacterial cellulose with glycerol, then with AP, followed by modification with a mixture of HSt at a concentration of 1% or 10% (as indicated by its number) and AP at a concentration of 60% (A40[S1.A60] and A40[S10.A60]), also showed a definite colour but were very susceptible to cracking. Once the materials with unfavourable attributes such as brittle texture or unpleasant tactile properties had been discarded, the following samples were subjected to further testing: A0, A40, A0[S10], and A0[S10.A60]. A comparative summary of the visual and sensory characteristics of the materials is presented in Table 1.

### 2.2. Morphological Characterisation of the Materials (SEM)

**Figure 2:**
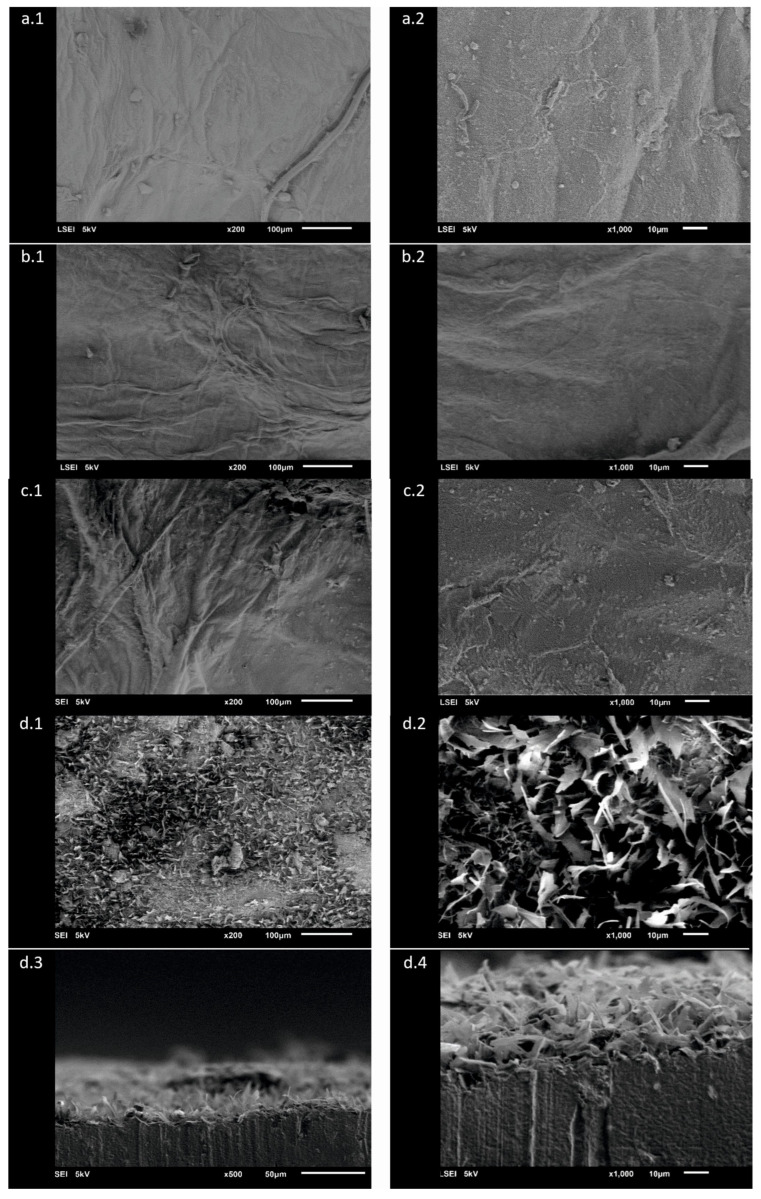
SEM images of materials. Images: (**a.1**,**a.2**)—bacterial cellulose (BC); (**b.1**,**b.2**)—bacterial cellulose modified with glycerol (A0); (**c.1**,**c.2**)— pre-treated BC modified with glycerol, then with apple powder at a concentration of 40%; (A40); (**d.1**–**d.4**)—bacterial cellulose modified with glycerol followed by modification with a mixture of HSt at a concentration of 10% and AP at a concentration of 60% (A0[S10A60]). Images (**a.1**–**d.2**) show views of the surface, whereas (**d.3**,**d.4**) show the cross-section.

The observation of the surface of the materials by means on the scanning electron microscopy revealed changes in their structure. The surface morphology of the analysed samples can be observed in the images presented in Figure 2. The SEM images of BC (Figure 2(a.1)) demonstrated the presence of fine cellulose fibres, which form a porous three-dimensional network structure. The overall morphological picture can be defined as a set of fibres organised into regions, with each region displaying a similar arrangement of fibres. Nevertheless, it is possible that the manner of organisation may vary between different regions. The compact cellulose cross-linked net displays a lattice structure comprising fibres of an apparently uniform size. In the more detailed Figure 2(a.2) (BC), it can be observed that the surface appears to be slightly wrinkled, but only to a slight extent, while maintaining a smooth appearance and uniformity.

The introduction of glycerol to a BC sample resulted in a noticeable morphological alteration, as evidenced by a visible flattening of wrinkles and a corresponding smoothing of the material’s surface. This is illustrated by comparing Figure 2(b.1,b.2). Additionally, the three-dimensional porous network structure of the BC becomes less discernible in comparison to the unmodified sample. The image of the material prepared by the introduction of apple powder into the cellulose displays the cellulose bundles as more visibly distinct, appearing thickened (Figure 2(c.1)). A more detailed examination of the surface in the magnified image indicates that it is also coated with apple powder (Figure 2(c.2)). Images of the material coated with a mixture of stearic acid and apple powder (Figure 2(d.1–d.4)) present an intriguing visual representation. The coating mixture forms a layer comprising interconnected flakes, which can be likened to wood chips in appearance. The flakes exhibit varying dimensions, yet they collectively cover the surface of the material with an even and uninterrupted coating. The cross-sectional images of A0[S10A60] (Figure 2(d.3,d.4)) permit the estimation of the thickness of the covering layer at an average of 17 µm. The samples used for imaging were first subjected to loads likely to be encountered during normal use, including pressure and friction. The images show that the coating formed by the HST and AP mixture remains stable under such mechanical stress.

### 2.3. Structural Analysis (FT-IR)

**Figure 3:**
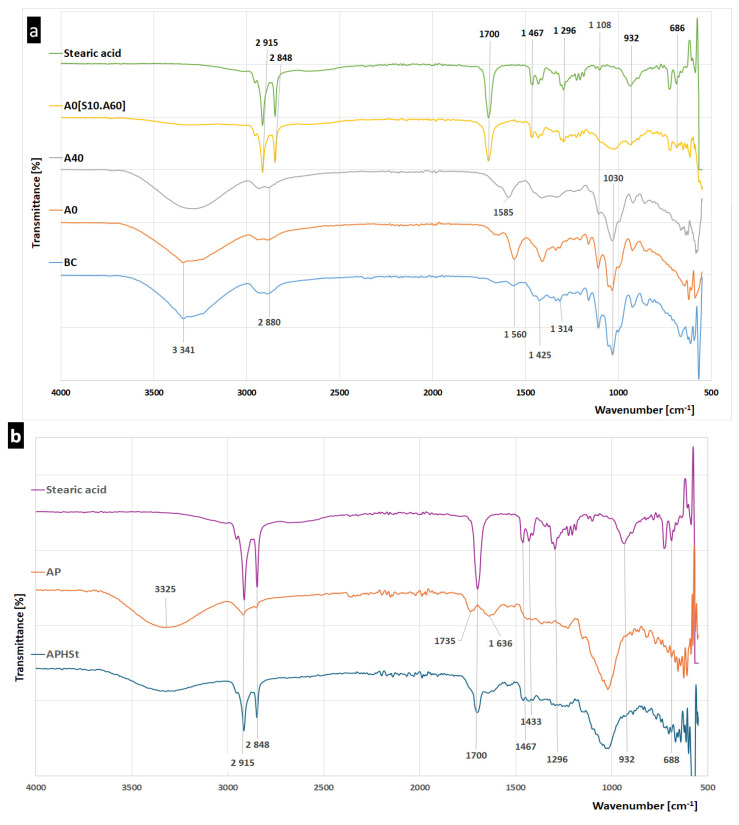
FTIR spectra of materials based on bacterial cellulose, apple powder, and stearic acid as their constituents. (**a**) shows material FTIR spectra, (**b**) shows coating layer component spectra. Samples codes: BC-pre-treated bacterial cellulose; A0 -BC modified with glycerol; A40 -pre-treated BC modified with glycerol, then with apple powder at a concentration of 40%; A0[S10.A60]- pre-treated BC modified with glycerol and covered with a mixture of stearic acid and apple powder; AP-apple powder; APHSt-the mixture of stearic acid and apple powder used as a hydrophobic coating.

Information regarding the functional groups and chemical bonds present in the materials was derived from Fourier-transform infrared spectroscopy (FT-IR) analyses. The chemical composition and structural and conformational differences between bacterial cellulose and its modified variants were analysed. The FT-IR spectra of pre-treated bacterial cellulose were compared with those of BC modified by glycerol and by entrapment in apple powder followed by coating in a combination of stearic acid and apple powder. The objective of gathering data regarding the structure of materials was to elucidate the nature of the alterations and to confirm the chemical modification of BC. The collected spectra are shown in Figure 3.

In the spectra of the materials, bands characteristic of cellulose and the modifying agent are visible. However, the applied modifications affected the position, width, shape, and intensity of some of those signals and led to the appearance of new peaks.

In the BC spectrum (Figure 3a), as well as in the AP spectrum (Figure 3b), carbohydrate-specific signals can be observed. Absorption bands characteristic of both the groups constituting the backbone of the molecule and the interactions between successive molecules in the structure were observed. In the functional group region, it is possible to observe the presence of O-H stretching vibrations, the absorption of hydroxyl groups of carbohydrate (a strong, broad band in the range of wave numbers 3600–2800 cm^−1^), C-H stretching of aliphatic CH_2_ groups (absorption bands at 2934–2882 cm^−1^), symmetric stretching and out-of-plane stretching of CH_2_ groups (1425 and 1314 cm^−1^) [32]. Furthermore, in the fingerprint region, detected signals include antisymmetric stretching of the C-O-C bridge of the β-1,4-glycosidic bond, C-C and C-O stretching vibrations, C-O-H bending, and skeletal vibrations of the C-O pyranose ring. The presence of hydrogen bonding (as evidenced by a peak at 3341 cm^−1^) indicates the existence of intramolecular connections (Figure 3a).

Changes in the structure of cellulose, a consequence of its modification during pre-treatment procedures; plasticization with glycerol (A0); and fusion with AP (A40) can be observed in the spectra. An intense band with a peak at 1560 cm^−1^ (A0) corresponds to antisymmetric carboxylate stretching bands (COO^−^) and resulted from cellulose oxidation with hydrogen peroxide. Further modification of this group, a change in band intensity, and a new peak (1585 cm^−1^) are evident in samples of AP-modified cellulose (A40). The change is probably due to the involvement of this group in the binding of the modifying component of the material.

In the spectra of BC and A0, an additional peak was identified at 3344 cm^−1^ in the broadband range of 3600–3000 cm^−1^. This peak can be assigned to the strength of the intramolecular O(3)H⋯O(5′) hydrogen bond [33]. However, this peak was not observed in A40 or in A40 [S10.A60]. Moreover, this is not the only notable change observed in the FTIR spectra in this wavenumber region of materials with AP as a component. Upon the introduction of AP to the material, the shape of the entire O-H bond-stretching band was observed to undergo a transformation, becoming more curved and indicating changes in the intensity value. The aforementioned observation is consistent with the findings of previous studies and may be associated with the interconnection of pectin and BC fibres through hydrogen bonding. [34]. Concurrently, in the range of 2930–2850 cm^−1^, the C-H bending bond demonstrates the presence of intramolecular bonds between cellulose and other components of the material [31]. The observed alterations in the BC spectra suggest that both modifiers were incorporated into the BC structure as a consequence of interactions between all material components, namely cellulose, glycerol, and pectin.

Hydrophobic APHSt coating prepared as a fusion of stearic acid and apple powder (Figure 3b) exhibits the presence of characteristic bands that can be attributed to the presence of the acid. In the stearic acid spectrum, the peaks observed at 2915 cm^−1^ and 2848 cm^−1^ can be attributed to symmetric and asymmetric stretching vibrations of -CH_3_, -CH_2_, and –CH. Furthermore, the carboxylic acid (C = O) stretching vibration is observed as strong bands near at 1700 cm^−1^. The peaks at 932 cm^−1^ and 1296 cm^−1^ correspond to -OH bending. The vibration in the region of 1258 cm^−1^ to 118 cm^−1^ represents CH_2_ wagging [35,36].

In the spectrum associated with APHSt (stearic acid and AP) and A0[S10.A60] (the material to which the coating layer was applied) a similar pattern was also observed. Comparing the spectrum of stearic acid with the spectra of the cover and materials changes such as signal weakening can be observed. The intensity of molecular vibration was markedly reduced in two regions. The first change concerns the broad band in the region from 3560 cm^−1^ to 3145 cm^−1^ assigned to O-H bond stretching (APHSt and A0[S10.A60]). The further affected bands were at 932 cm^−1^ and 1296 cm^−1^ (Figure 3b), suggesting interactions with AP influencing -OH bending vibrations. In the spectrum of A0[S10.A60], the introduction of the coating resulted in a weakened signal in the region from 1080 cm^−1^ to 980 cm^−1^, with the absorption peak at 1030 cm^−1^, which is attribute to the bending of C–O–H bonds of carbohydrates [37,38,39] or C–O–C pyranose ring skeletal vibration [37,40,41].

### 2.4. Wettability

The surface wettability of the materials was studied based on the observation of changes of deionized water and diiodomethane droplet geometry. Table 2 shows the values of the measured contact angles for samples BC, A0, A40, and A0[S10.A60]. The unmodified bacterial cellulose has the strongest hydrophilic character. The addition of a modifier in the form of glycerol in the A0 sample leads to only a slight increase in the water contact angle. In turn, in sample A40, the nature of the surface changes—in this case the modification led to its hydrophobization. The value of the contact angle increased by almost twice, and the difference was statistically significant. The value of the contact angle reached the commonly considered limit value for hydrophilic surfaces. Modification with the mixture of stearic acid and apple powder, A0[S10.A60], significantly enhanced the contact angle of the material and reached 124.5°. This phenomenon can be explained by the large amount of introduced -CH_2_- and -CH_3_ groups originating from the long hydrocarbon chain of stearic acid. The hydrophobicity obtained in material A0[S10.A60] is identical to that obtained by modification of BC with carboxymethyl cellulose and beeswax at a concentration of 40%, modifiers that also belong to the group of natural and biodegradable raw materials [27]. The physical entrapment of soy protein isolate and glycerol or fungal protein and glycerol in BC is another example of the hydrophobization of BC in combination with natural raw materials. The water resistance of these materials was found to be approximately 110° [42]. It should be noted that the provided examples are not representative of the majority of BC modifications that utilise plant-based raw materials. In the general case, these modifications achieve water contact-angle values of approximately 80° [43].

The values of the surface free energy and its disperse and polar components are also shown in Table 1. The polar-component SEP is the sum of the forces of hydrogen, acid—base, and inductive interactions, while the dispersion component determines the size of intermolecular interactions called London forces. Dispersion interactions always occur, while polar interactions occur only when polarization of chemical bonds takes place [44]. The share of polar interactions is the highest in the unmodified cellulose sample and in the A0 sample. Samples modified with stearic acid had an extremely low value of 0.1 mJ/m^2^ of the polar component. In this case, the SEP value is also the lowest, which means that for this sample, the surface has the lowest adhesion potential. In turn, samples BC and A0 achieved the highest SEP values of 95.2 and 54.8 mJ/m^2^, respectively, and sample A40 reached an intermediate value between BC and A0[S10A60].

The surface of the materials displays a range of roughness, from a very low level (Ra 0.3 µm) to a high level (Ra 4.6 µm). The BC roughness values indicate that the surface is smooth, which is consistent with expectations, given the high degree of ordering of the cellulose fibres. The introduction of glycerol and AP to BC led to an observable elevation in the surface’s irregularity, resulting in a notable increase in its overall roughness. This effect manifested similarly across both materials A0 and A40 and was statistically significant when compared to BC. The coating layer in the material significantly reduced the roughness (A40 vs. A0[S10A60]). Surface roughness does not appear to be the most significant factor in the hydrophilicity or hydrophobicity of these materials (Pearson coefficient of −0.1).

### 2.5. Antimicrobial Activity

The antimicrobial activity of BC, A0, A40, and A0[S10.A60] was tested against strains of microorganisms belonging to a Gram-negative bacterium (*Escherichia coli*) and Gram-positive bacteria (*Bacillus subtilis* and *Staphylococcus aureus*), mould (*Aspergillus niger*), and yeast (*Candida albicans*). Selected microorganisms belong to different taxonomic groups and are characterised by different growth physiologies and sensitivities to disinfectants. Table 3 provides a summary of the findings from the evaluation of the antimicrobial properties of the selected materials in relation to specific microorganisms.

It was found that the materials, bacterial cellulose modified with glycerol, glycerol and AP or glycerol, and AP followed by covering with a mixture of HSt and AP, exhibited antibacterial properties comparable to those of BC when tested on cultures of microorganisms, including *E. coli*, *S. aureus*, and *B. subtilis*. No adverse effects on the growth of bacteria were observed when any of the tested materials were employed. No inhibition zone was identified, and bacteria continued to proliferate beneath the samples. Similarly, fungal growth did not indicate the presence of an inhibition zone in any of the samples. The formation of a zone of growth inhibition was not observed for any of the test materials, nor was increased microbial growth observed in this zone around the materials. This can be interpreted as the absence of migration of material modifiers into the culture medium. Antimicrobial activity was observed in tests with fungal strains; the growth was limited underneath two of the four materials: A0 and A40. Both of those materials were obtained as a consequence of modifications conducted using either glycerol alone (A0) or a combination of glycerol and AP (A40). This observation is consistent with the literature data; in the work of M. Rouhi et. all., the authors observed intense activity against *S. aureus*, *E. coli*, and *C. albicans* and a weak antimicrobial effect on *Pseudomonas aeruginosa* for films made using either glycerol or polyvinyl alcohol reinforced with glycerol, bacterial cellulose nanocrystals [45].

The antimicrobial effect of A0[S10A60] was different from what was expected. Stearic acid was chosen as a coating agent to give the materials additional antimicrobial properties. This was based on observations in nature and research results. It is well known fact that fatty acids are produced by plants and algae to defend themselves against pathogens, including multidrug-resistant bacteria (MDRB) [46]. The literature contains a substantial number of experimental studies that confirm the antibacterial effect of fatty acids (both saturated and unsaturated), such as lauric acid, capric acid, palmitic acid, and stearic acid [47]. Moreover, some researchers consider them to be a promising option for the development of next-generation antibacterial agents to treat a broad spectrum of bacterial infections. The most probable explanation for the absence of the considered antimicrobial characteristics is the insufficient concentration of HSt to manifest these properties concurrently with a substantial quantity of AP in the coating.

The tested materials were found to have no adverse impact on or demonstrate a low level of activity with respect to the growth of the selected strains of microorganisms. Consequently, their use in certain contexts, such as in food protection, is not currently limited. However, the fundamental assumption of biodegradability has been presented, and it can be assumed that the materials will not pose a burden on the environment after use.

### 2.6. Binding Bacterial Cellulose and Pectin—Modelling Interactions

In this study, the AP was selected as a main BC modifier. Fruit pomace is mainly composed of cell wall polysaccharides, i.e., fibre components such as cellulose, pectin, and hemicelluloses [48]. The predominant component of fruit pomace is cellulose, with an average content of about 43%, followed by hemicellulose, whose content varies from about 20% to 32% [49]. The content of pectin also varies and is influenced by the source and the conditions used during the preparation process. In AP, it was found to be 10.91 %. Pectin is a structural heteropolysaccharide composed of two basic structures: a linear homopolymer, homogalacturonan I (HG), and a side-chained polymer [49]. HG is a chain of galacturonic acid residues with α-(1–4) linkages, some of which are methyl-esterified. The second region is a complex structure composed of a backbone in which the main chain includes repeats of α-(1-4)-L-galacturonic acid and α-(1-2)-L-rhamnopyranose residues, which are highly substituted by polysaccharides such as arabinan, galactan, or arabinogalactan [50].

The physicochemical properties of the BC and its modifiers, including homogalacturonan and rhamnogalacturonan (RG), were verified, and the mechanisms of their mutual interaction were determined. This was achieved through the implementation of quantum chemistry and physics tools. A detailed analysis of the systems containing isolated BC, HG, and RG was conducted, and the corresponding energies were determined to be −338 kcal/mol, −1364 kcal/mol, and −2804 kcal/mol, respectively (see Table 4). For the combined systems, comprising BC and one modifier, the respective energies were found to be −710 kcal/mol (BC with HG) and −7158 kcal/mol (BC with RG).

**Figure 4:**
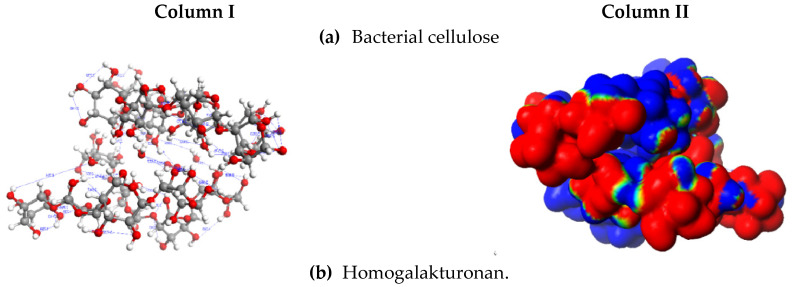

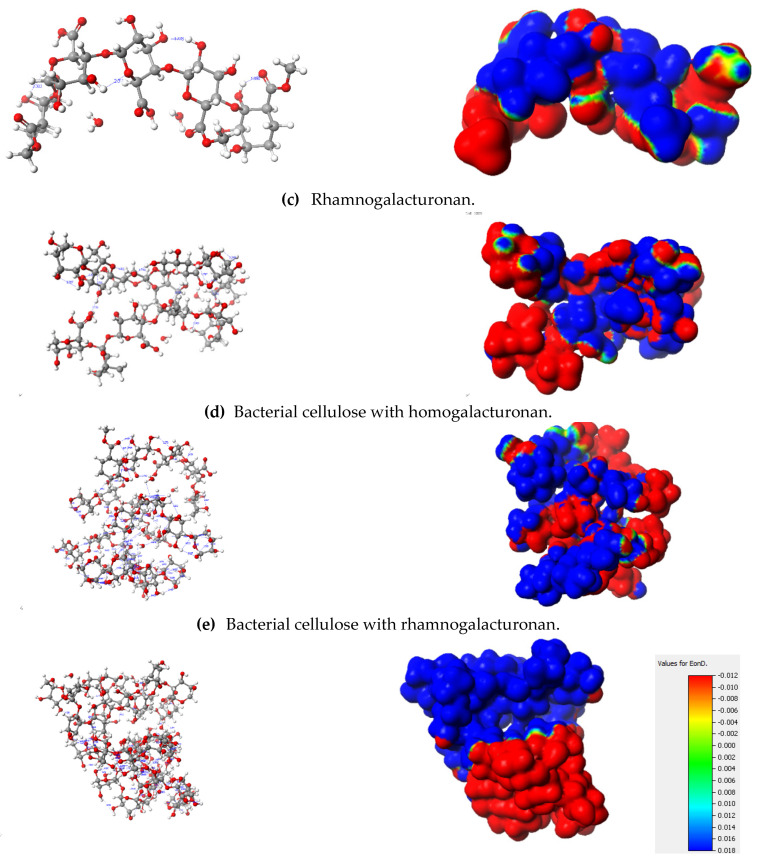
Hydrogen bond map (**column I**) with an example electron distribution (**column II**) of the modelled structures.of polysaccharides (**a**–**c**) and composite systems of bacterial cellulose–polysaccharide (**d,e**).

The reduction in energy observed in cellulose–polysaccharide composite systems with HG and RG polymers is indicative of their thermodynamic stabilization following the formation of the mixed biopolymer system. This outcome is observed to arise as a consequence of the operation of two distinct synergistic mechanisms. The first mechanism is associated with the mutual polarity of cellulose and the pectin fraction to which it is being applied. This can be described as the interaction of dipole systems, with mutual orientation observed (Figure 4d,e). The contribution of dispersive forces [51] and electrostatic interactions, in addition to hydrogen bonding between OH^…^O groups [52,53,54], to the maintenance of the internal cohesion of cellulose microfibrils and the sorption of non-cellulosic substances was highlighted as being of significant importance [55].

The second interaction mechanism is related to the generation of additional hydrogen interactions at the phase boundary. This causes the systems to exhibit high adhesion and flexibility. The system does not stiffen because no chemical bonds are formed; however, physical bonds are observed, which can be broken and regenerated relatively easily. The formation of additional hydrogen bonds occurs between groups that can readily alter their conformation and fit into the spatial arrangement of cellulose due to their low energy requirements. These groups are situated at the extremities of the chains (Figure 4d,e). These substructures do not necessitate a conformational alteration of the macromolecules; rather, it is sufficient for the fragments of these macromolecules, comprising one to two carbon atoms with oxygen and hydrogen atoms, to undergo such a change. Furthermore, the modelling demonstrated that the generation of physical interactions at the interface (dipole–dipole interactions and hydrogen bond formation) resulted in the removal of some water molecules from the macromolecules. These findings support the observations derived from the experimental determination of the hydrophobicity of materials. The measured water contact angle of A40, the material with AP, was 83.1, whereas without the modifying agent (A0) it was 44.0, confirming the change in the material’s character to become much more hydrophobic.

The presence of water molecules adsorbed from the air was observed to interfere with the process of maximizing the interaction between cellulose and the corresponding pectin (Figure 4e). The empirical data obtained from FTIR analysis also demonstrates alterations in hydrogen bonding, which resulted in a weakening of the interactions between cellulose and water in the presence of pectin. Hydrogen bonding from cellulose with pectin carbonyls as acceptors was suggested in [56]. The thermodynamic parameters of hydrogen bonds between cellulose and water indicate competition with hydrogen bonds between cellulose and cellulose or between cellulose and another polymer [57,58].

## 3. Materials and Methods

### 3.1. Reagents

In the experimental work, hydrogen peroxide (H_2_O_2_ 30.0 ± 1.0%, Chempur, Piekary Śląskie, Poland), sodium hydroxide, (NaOH pellets Merck Sp. z o.o., Warszawa, Poland), acetic acid (CH_3_COOH 99.5–99.9%, Avantor Performance Materials, Poland), glycerol (Chempur, Poland), Ethanol (96%, StanLab, Lublin, Poland), stearic acid (Merck Life Science Sp.z.o.o. Poznan; Poland), and diiodomethane (CH_2_I_2_ 99%, CAS Number 75-11-6, SigmaAldrich, Saint Louis, MO, USA) were used. All the reagents were used without further purification. The pellicle was provided by Kameleon Kulinarny Joanna Grzybowska Łódź (Poland). Powder of apple fibre (100% micronized apple fibre, food grade) was purchased from Aura Herbals sp. z o.o., Gdansk, Poland. Bacterial media, Tryptic Soy Agar (TSA) with pH ranging from 7.1 to 7.5, were obtained from Merck KGaA (Darmstadt, Germany). The medium used in the yeast and mould experiments was Malt Extract Agar (MEA, from Merck KGaA). The pH of the medium ranged from 5.4 to 5.6.

### 3.2. Production of Materials

#### 3.2.1. Production of Biopolymer

The biopolymer was obtained in a bioconversion process using SCOBY. A detailed account of the procedure has already been provided. [31]. The symbiotic culture was cultivated in a sweetened infusion of black tea for approximately three weeks. Static experimental conditions with minimal illumination were maintained throughout this period. Once the pellicle of the bacterial cellulose reached a thickness of approximately 1–2 cm, the process was terminated. The resulting biopolymer layer formed on the surface of the solution was then gently removed from the growth medium.

#### 3.2.2. Bacterial Cellulose Preparation

The biopolymer obtained from the fermentation process was pre-treated to prepare bacterial cellulose and its further modifications. The detailed procedure has been presented previously [31]. The main steps of the pre-treatment included washing, bleaching, and swelling. Washing aimed to rinse out the remaining growing solution, as well as to remove the microorganisms, and was achieved by washing the pellicle in NaOH solution (3%), followed by washing in acidic solution (acetic acid, pH 3.0) and bleaching with H_2_O_2_ solution (5%). After removal of the hydrogen peroxide solution, the biopolymer was swollen with an NaOH solution (8%) in an ultrasonic bath (Sono Swiss Sw12H; Switzerland). At the end of the pre-treatment procedure, the BC was subjected to a washing process with tap water until a neutral pH was attained.

#### 3.2.3. Bacterial Cellulose Modification with the Apple Powder

BC modification by entrapping apple powder in the polymer was performed following the procedure described previously [31], an adaptation of a modification method presented by Kim et al. [42]. The modifying mixtures were prepared by combining the apple powder suspension with glycerol as a plasticizer. The concentration of AP in the subsequent mixture variants ranged from 10 to 60% by weight of BC, each of which was prepared by suspension of the powder in water (the weight of water was 10 times the weight of BC) and standardisation by homogenisation. After the addition of glycerol (30 wt %), the mixtures were first homogenised for a further 2 min, then ultrasonicated for 30 min at 25 °C. After adjusting the pH to 10.0 (±0.5) with 1M NaOH solution, the mixtures were incubated at 80 °C in a water bath with shaking at 70 rpm for 20 min. The BC was then immersed in the modifying mixture to ensure that it was completely covered and ultrasonicated for 30 min at 25 °C. The modification was continued for 1 h in a water bath (35 °C), shaking at 80 rpm. Finally, BC modified at different concentrations of AP was dried for 4 days under mild conditions at 35 °C in a laboratory oven.

### 3.3. Surface Modifications

The surface of BC or the materials was coated with either stearic acid or a mixture of HSt and AP. Four ethanolic solutions of HSt were prepared using 99.9% ethanol to reach final concentrations of 1%, 3%, 5 %, and 10 %. Solutions of extreme concentrations, i.e., 1% and 10%, were divided into two bottles with the same concentration, and AP was added to one half to obtain a concentration of 60%. Samples of materials were immersed in an ethanolic solution or suspension of AP and HSt, and the bottles were closed to avoid evaporation. After 24 h of incubation in the water bath at 60 °C, samples of materials were removed and transferred to an oven. All the materials were dried for 24 h at 30 °C, and the extra powder of the dry samples was removed. The variants of the modifications are summarized in Table 5.

### 3.4. Fourier Transform-Infrared Spectroscopy (FT-IR)

The analyses were conducted using a Nicolet 6700 (Thermo Scientific, Waltham, MA, USA) Fourier transform infrared (FT-IR) spectrometer. The measurement region spanned 4000 to 600 cm⁻^1^, with a spectral resolution of 2 cm⁻^1^ and 64 scans per sample. Background spectra were obtained prior to each measurement. Spectral data were processed using OMNIC ver. 8.0 (Thermo Fisher Scientific Inc., Waltham, MA, USA).

### 3.5. Surface Wettability

The measurement was carried out using an EasyDrop FM40 contact-angle measuring system (Krüss GmbH, Germany). The detailed droplet geometry was analysed by means of specialized EasyDrop software (Drop Shape Analysis 1.90.0.14 software (Krüss GmbH, Hamburg, Germany). Wetting measurement was conducted in an ambient environment (22 ± 1 °C air humidity: 50 ± 5%). The study used the sessile drop method (volume: 0.8 µL). Measurements were repeated in at least 5 randomly selected locations.

The wettability of the materials was measured using the sessile drop technique for two liquids of different polarity and known surface tension, i.e., distilled water and diiodomethane. The wettability measurements were subsequently complemented by an assessment of the surface free energy (SFE) based on the Owens–Wendt theoretical model. The Owens–Wendt (also Kaelble–Owens–Wendt) method involves determining the dispersive and polar components of the SFE based on the Bethelot hypothesis. In order to ascertain the SFE, it is necessary to utilise two distinct liquids, namely water (which is polar in nature) and diiodomethane (which is dispersive in character), both of which have been previously characterised in terms of their surface tension and the polar and dispersive components thereof. As a highly polar liquid, distilled water has been employed due to the fact that its polar component is 51 mN/m and the dispersive component is 21.8 mN/m. The second liquid was diiodomethane, which exhibited the following SFE components: polar—2.4 mN/m; dispersive—48.6 mN/m [59].

$$\gamma_{L}=\gamma_{L}^{d}+\gamma_{L}^{p}\;\gamma_{S}=\gamma_{S}^{d}+\gamma_{S}^{p}\;1+\cos\Theta =\frac{2}{\gamma_{L}}\left[{\left(\gamma_{L}^{d}\gamma_{S}^{d}\right)}^{\frac{1}{2}}+{\left(\gamma_{L}^{p}\gamma_{S}^{p}\right)}^{\frac{1}{2}}\right]$$

where: γL is the free surface energy of the liquid in equilibrium with the saturated water vapour of the liquid; γS is the free surface energy of the solid in equilibrium with the saturated vapour; γdL and γpL are the dispersive and polar components of measuring liquid’s surface energy, respectively; γdS and γpS are the dispersive and polar components of SFE of the examined materials, respectively [60].

### 3.6. Scanning Electron Microscopy (SEM)

The structure and morphology of the of BC and materials were examined by a JEOL JSM-6610LV scanning electron microscope working in both high- and low-vacuum modes. Prior to the examination, samples were covered with 5 nm-thick layer of gold deposited by means of the magnetron sputtering method.

### 3.7. Antimicrobial Activity Tests

The antimicrobial activity of the materials was tested against *Staphylococcus aureus* (ATCC 6538), *Escherichia coli* (ATCC 8739), *Bacillus subtilis* (ATCC 6633), *Candida albicans* (ATCC 10231), and *Aspergillus niger* (ATCC 16404) microorganisms. The microorganisms used in the study were obtained from the American Type Culture Collection via Microbiologics (Grenoble Cedex, France). The strains were stored on TSA (bacteria) and MEA (yeast and moulds) slants at 4 °C. Prior to each experiment, the bacterial and fungal strains were activated on TSA and MEA medium, respectively. The cultures were incubated at 37 °C for 24–48 h (bacteria and yeast) and 30 °C for 24–48 h (*B. subtilis*) or 5 days (moulds); then, an inoculation suspension was prepared.

The antimicrobial activity of materials was assessed using a qualitative agar diffusion plate method according to the modified methodology described in PN-EN ISO 20645:2006). Two parallel rows of inoculum of the test microorganisms were applied to plates containing the respective media at a density of 1–2 × 10^6^ cfu/cm^3^ (for fungi) or 1–2 × 10^8^ cfu/cm^3^ (for bacteria). Material samples were then placed perpendicular to the growth lines using sterile tweezers and incubated. Microorganisms were cultured on a dedicated medium under specific temperature and time conditions (bacteria were cultured on TSA medium at 37 ± 2 °C for 24–48 h, except B. subtilis, which incubated at 30 ± 2 °C; yeasts were cultured on MEA medium at 37 ± 2 °C for 48 h; and moulds were cultured on MEA medium at 30 °C for 5–7 days). The nature of growth was observed, and zones of growth inhibition were measured. Microbial control samples were also inoculated. The method determined zones of growth inhibition under and around the textile sample according to the scale shown in Table 6.

### 3.8. Pectin Content Determination

The pectin content of the apple pomace was determined using a method originally proposed by V. Chandel et al. [61]. The extraction of pectin was conducted using an autoclave at a temperature of 121° for a period of 60 min, followed by precipitation using ethanol.

### 3.9. A Model System of Binding Bacterial Cellulose and Pectin

The mechanisms of chemical interactions between bacterial cellulose and selected components of apple powder, as well as their potential influence on the physico-chemical properties of the materials, were determined using tools from chemistry and quantum physics. A model system for the interaction between cellulose and homogalacturonan or rhamnogalacturonan was created using SCIGRESS v.FJ 2.7 software. The theoretical analyses were conducted with the assumption that the compounds under investigation had been isolated from their natural environment, which allowed for the exclusion of any additional external interactions. The exception to this was the consideration of potential interactions with water molecules. The presence of these molecules was accounted by the well-established capacity of cellulose to absorb water, including that contained in air. As the molecules comprise only three types of atoms (O, H, and C), a semi-empirical method was employed, which permitted relatively rapid calculations.

The application of semi-empirical techniques enabled the determination of changes in FTIR spectra, both for pure substances and their mixtures. Subsequently, changes in the energy values of the systems were characterised. Furthermore, quantitative alterations in hydrogen bonds were mapped out. Based on the modelled structures, analyses of the electron distribution on their surface were conducted to illustrate the potential for the formation of dipole systems as a means of interaction for the biopolymers under investigation.

### 3.10. Surface Roughness

An analysis of the surface roughness of the samples was performed with a Hommel-Etamic T1000 profilometer (Jenaoptik Industrial Metrology, Jena, Germany) and EVOVIS software (Jenaoptik Industrial Metrology, Jena, Germany) (version 2.00.1.00). The surface roughness was determined as average roughness (Ra) [µm], average maximum surface height (Rz) [µm], and the maximum of all per-sampling-length peak-to-valley heights (RMax) [µm] according to the evaluation procedure defined in ISO 4288: 1997 [62].

### 3.11. Statistical Analysis

Statistical analysis of the data was performed using R computational language [63]. (R Core Team (2022). R: A language and environment for statistical computing. R Foundation for Statistical Computing, Vienna, Austria. URL). For post hoc tests, the package called Agricolae: Statistical Procedures for Agricultural Research (version 1.3-5) was used [64]. The data were analysed using a one-way ANOVA (*p* < 0.05) followed by multiple comparisons using Tukey’s test, with significance set at *p* < 0.05.

## 4. Conclusions

Bacterial cellulose (BC) is a promising biopolymer with significant potential for the development of innovative products and applications. Its properties align well with current research trends. Numerous studies have demonstrated that BC can be modified in a wide range of ways, imparting desired functionalities and properties while maintaining an appropriate quality profile. New research findings continue to demonstrate its versatility in various technological applications. The primary objective of this study was to develop a material exhibiting hydrophobic characteristics that are contrary to the intrinsic properties of bacterial cellulose. Such a material would align with the current trends and market demand for biodegradable, naturally derived materials, offering a potential alternative to the fabrication of superhydrophobic surfaces using toxic and/or expensive materials. The proposed modifications will allow BC to exhibit these desired properties. The modification involved the use of waste material derived from the juice production process, specifically, apple powder and natural waxes such as stearic acid. This objective was successfully achieved. The incorporation of natural wax is likely to enhance the resistance of material containing it to microbial attack due to the inherent antimicrobial properties of waxes. Traditionally, wax barrier coatings are a natura defence mechanism that also protects the fruit from microbial attack, which is what nature uses it for. The proposed modification does not have such an antimicrobial effect, presumably due to the high AP content of the whole material. In the future, such properties could also be obtained by soaking the whole material in a suitable agent whose properties are tailored to the intended purpose.

## Figures and Tables

**Table 1:** Sensory characteristics of materials or groups of materials.

Material	Sensory Characteristics		
	Colour Uniformity	Tactile Sensation	Flexibility and Fracture Resilience
BC	Darker, patchy discolouration, semitransparent	Smooth, dry	Flexible
A0	Uniform, semitransparent	Smooth, slightly oily	Flexible
A0[S1], A0[S3], A0[S5], A0[S10],	Uniform, opaque	Dry, nonslip feel upon touch	Crumbling and cracking
A40	Areas of discolouration, opaque	Smooth, slightly slippery but pleasant to the touch	Flexible
A40[S1], A40[S3], A40[S5], A40[S10]	Uniform, opaque	Dry, rough	Cracking when bent
A0[S1.A60], A0[S10.A60]	Areas of discolouration, opaque	Slightly slippery, smooth	Flexible
A40[S1.A60], A40[S10.A60]	Darker, patchy discolouration, opaque	Dry, rough, perceptible unevenness	Flexible, susceptible to cracking

Samples codes: BC—bacterial cellulose (biopolymer after fermentation process pre-treated); A0—bacterial cellulose modified with glycerol; A0[S1], A0[S3], A0[S5], and A0[S10]—bacterial cellulose modified with glycerol followed by HSt at a concentration indicated by the bracketed numbers; A40—bacterial cellulose modified with a mixture of glycerol and AP at a concentration indicated by the number; A40[S1], A40[S3], A40[S5], and A40[S10]—bacterial cellulose modified with a mixture of glycerol and AP followed by modification with HSt at a concentration indicated by the bracketed numbers; A0[S1.A60] and A0[S10.A60]—bacterial cellulose modified with glycerol followed by modification with a blend of HSt at a concentration of 1% and 10%, respectively (indicated by the respective numbers), and AP at a concentration of 60%; A40[S1.A60] and A40[S10.A60]—bacterial cellulose modified with a mixture glycerol and AP followed by modification with a mixture of HSt at a concentration of 1% and 10%, respectively (indicated by the respective numbers), and AP at a concentration of 60%.

**Table 2:** Water and diiodomethane contact angle, surface free energy with dispersive and polar components, and Roughness, where: Ra is average roughness, Rz is average maximum surface height, RMax is maximum of all per-sampling-length peak-to-valley heights.

	Material			
	BC	A0	A40	A0[S10.A60]
Water contact angle [°]	38.0 ± 3.4 ^c^	44.0 ± 7.5 ^c^	83.1 ± 5.0 ^b^	124.5 ± 2.4 ^a^
	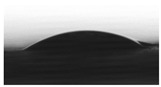	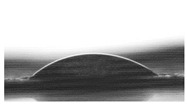	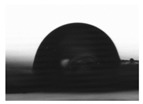	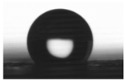
Diiodomethane contact angle [°]	69.9 ± 2.0 ^b^	74.3 ± 8.5 ^b^	76.0 ± 2.2 ^b^	101.1 ± 3.7 ^a^
	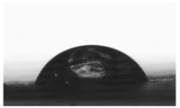	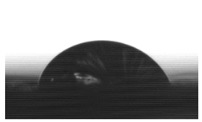	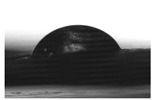	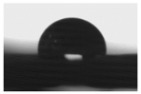
Polar component [mJ/m^2^]	47.5 ± 1.3	44.7 ± 1.7	10.3 ± 1.9	0.1 ± 0.9
Dispersive component [mJ/m^2^]	11.6 ± 0.7	10.1 ± 1.7	14.7 ± 0.8	8.3 ± 1.4
Surface Free Energy [mJ/m^2^]	59.2 ± 0.9 ^a^	52.7 ± 1.7 ^b^	25.0 ± 1.4 ^c^	8.4 ± 1.2 ^d^
Roughness				
Ra [µm]	0.3 ± 0.0 ^b^	4.6 ± 1.7 ^a^	4.6. ± 0.9 ^a^	1.2 ± 0.2 ^b^
Rmax [µm]	2.3 ± 0.2 ^b^	37.7 ± 11.5 ^a^	32.1 ± 8.3 ^a^	8.5 ± 1.7 ^b^
Rz [µm]	1.8 ± 0.2 ^b^	21.9 ± 7.6 ^a^	21.7 ± 6.9 ^a^	6.4 ± 1.4 ^b^

Values with the same superscript letter (a, b, c, d) are not significantly different (*p* > 0.05) according a one-way ANOVA (*p* < 0.05) followed by multiple comparisons using Tukey’s test. The results are presented as an average (n = 5) ± standard deviation. Sample codes: BC—pre-treated bacterial cellulose; A0—BC modified with glycerol; A40—BC pre-treated modified with glycerol and then with apple powder at a concentration of 40%; A0[S10.A60]—BC pre-treated followed then modified with glycerol and covered with the mixture of stearic acid and apple powder. Ra—average roughness, Rz—average maximum surface height, RMax maximum of all per-sampling-length peak-to-valley heights.

**Table 3:** The antibacterial effects of materials on *E. coli* (Ec), *B. subtilis* (Bs), *S. aureus* (Sa), *C. albicans* (Ca), and *A. niger* (An).

	Material			
Strain	BC	A0	A40	A0[S10A60]
	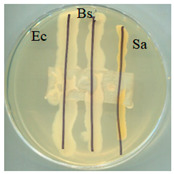	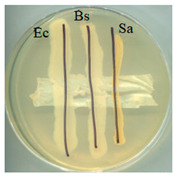	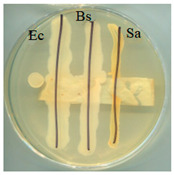	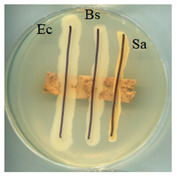
	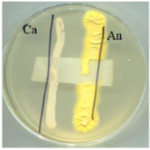	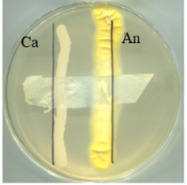	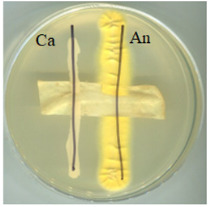	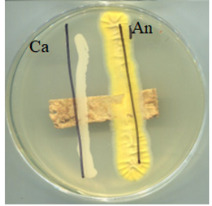
*E. coli* (Ec)	-	-	-	-
*S. aureus* (Sa)	-	-	-	-
*B. subtilis* (Bs)	-	-	-	-
*C. albicans* (Ca)	-	+	+	-
*A. niger* (An)	-	+	+	-

Materials: BC—pre-treated bacterial cellulose; A0—BC modified with glycerol; A40—pre-treated BC modified with glycerol, then with apple powder at a concentration of 40%; A0[S10A60]—pre-treated BC modified with glycerol and covered with the mixture of stearic acid and apple powder; Ec—*E. coli*; Sa—*S. aureus*; Bs—*B. subtilis*; Ca—*C. albicans*; An—*A. niger*.

**Table 4:** The energy of cellulose and polysaccharide components of pectin and combined systems.

Compound	Chemical Composition			Energy, E [kcal/mol]	Number of Hydrogen Bonds
	C	O	H		
Cellulose	80	92	152	−4338	48
Homogalakturonan	35	30	56	−1364	4
Ramnogalakturonan	75	62	126	−2804	12
Cellulose with Homogalakturonan	115	122	208	−5710	97
Cellulose with Ramnogalakturonan	155	154	278	−7158	82

**Table 5:** The materials prepared by bacterial cellulose modifications.

	Material Code	Sample Composition			
		Core		Coating	
		Glycerol [%]	Apple Powder [%]	Stearic Acid [%]	Apple Powder [%]
1	BC	0	0	0	0
2	A0	30	0	0	0
3	A0[S1]	30	0	1	0
4	A0[S3]	30	0	3	0
5	A0[S5]	30	0	5	0
6	A0[S10]	30	0	10	0
7	A40	30	40	0	0
8	A40[S1]	30	40	1	0
9	A40[S3]	30	40	3	0
10	A40[S5]	30	40	5	0
11	A40[S10]	30	40	10	0
12	A0[S1.A60]	30	0	1	60
13	A0[S10.A60]	30	0	10	60
14	A40[S1.A60]	30	40	1	60
15	A40[S10.A60]	30	40	10	60

Samples codes: BC—bacterial cellulose (pre-treated biopolymer after fermentation process); A0—bacterial cellulose modified with glycerol; A0[S1], A0[S3], A0[S5], and A0[S10]—bacterial cellulose modified with glycerol followed by HSt at a concentration indicated by the bracketed numbers; A40—bacterial cellulose modified with a mixture of glycerol and AP at a concentration indicated by the number; A40[S1], A40[S3], A40[S5], and A40[S10]—bacterial cellulose modified with a mixture of glycerol and AP followed by modification with HSt at a concentration indicated by the bracketed numbers; A0[S1A60] and A0[S10.A60]—bacterial cellulose modified with glycerol followed by modification with a mixture of HSt at a concentration of 1% and 10%, respectively (indicated by the respective numbers), and AP at a concentration of 60%; A40[S1.A60] and A40[S10.A60]—bacterial cellulose modified with a mixture glycerol and AP followed by modification with a mixture of HSt at a concentration of 1% and 10%, respectively (indicated by the respective numbers), and AP at a concentration of 60%.

**Table 6:** Descriptive scale of growth characteristics.

Growth Inhibition Zone [mm]	Growth Characteristics	IMPACT	
>1	Inhibition of growth around and under the sample.	Very good activity	+++
0–1	Inhibition of growth around and under the sample.	Good activity	++
0	The sample did not produce a zone of inhibition and did not inhibit growth.		
0	No zone of growth inhibition, slight growth under the sample.	Low activity	+
0	There was no zone of growth. inhibition observed, but growth was detected under the sample.	Lack of activity	-

## Data Availability

Data are contained within the article.

## References

[1] Zheng J., Suh S. (2019). Strategies to Reduce the Global Carbon Footprint of Plastics. Nat. Clim. Chang..

[2] Cruz M.A., Flor-Unda O., Avila A., Garcia M.D., Cerda-Mejía L. (2024). Advances in Bacterial Cellulose Production: A Scoping Review. Coatings.

[3] Jayabalan R., Malbaša R.V., Lončar E.S., Vitas J.S., Sathishkumar M. (2014). A Review on Kombucha Tea-Microbiology, Composition, Fermentation, Beneficial Effects, Toxicity, and Tea Fungus. Compr. Rev. Food Sci. Food Saf..

[4] Esa F., Tasirin S.M., Rahman N.A. (2014). Overview of Bacterial Cellulose Production and Application. Agric. Agric. Sci. Procedia.

[5] Lahiri D., Nag M., Dutta B., Dey A., Sarkar T., Pati S., Edinur H.A., Kari Z.A., Noor N.H.M., Ray R.R. (2021). Bacterial Cellulose: Production, Characterization, and Application as Antimicrobial Agent. Int. J. Mol. Sci..

[6] Rastogi A., Banerjee R. (2020). Statistical Optimization of Bacterial Cellulose Production by Leifsonia Soli and Its Physico-Chemical Characterization. Process Biochem..

[7] Abitbol T., Rivkin A., Cao Y., Nevo Y., Abraham E., Ben-Shalom T., Lapidot S., Shoseyov O. (2016). Nanocellulose, a Tiny Fiber with Huge Applications. Curr. Opin. Biotechnol..

[8] Campano C., Balea A., Blanco A., Negro C. (2015). Enhancement of the Fermentation Process and Properties of Bacterial Cellulose: A Review. Cellulose.

[9] Wang Z., Li S., Zhao X., Liu Z., Shi R., Hao M. (2025). Applications of Bacterial Cellulose in the Food Industry and Its Health-Promoting Potential. Food Chem..

[10] Infante-Neta A.A., D’Almeida A.P., Albuquerque T.L.D. (2024). Bacterial Cellulose in Food Packaging: A Bibliometric Analysis and Review of Sustainable Innovations and Prospects. Processes.

[11] Blanco Parte F.G., Santoso S.P., Chou C.C., Verma V., Wang H.T., Ismadji S., Cheng K.C. (2020). Current Progress on the Production, Modification, and Applications of Bacterial Cellulose. Crit. Rev. Biotechnol..

[12] Rajwade J.M., Paknikar K.M., Kumbhar J.V. (2015). Applications of Bacterial Cellulose and Its Composites in Biomedicine. Appl. Microbiol. Biotechnol..

[13] Stumpf T.R., Yang X., Zhang J., Cao X. (2018). In Situ and Ex Situ Modifications of Bacterial Cellulose for Applications in Tissue Engineering. Mater. Sci. Eng. C.

[14] Ludwicka K., Rytczak P., Kołodziejczyk M., Gendaszewska-Darmach E., Chrzanowski M., Kubiak K., Jędrzejczak-Krzepkowska M., Bielecki S. (2016). Bacterial Nanocellulose—A Biotechnological Product for Biomedical Applications. New Biotechnol..

[15] Klemm D., Schumann D., Udhardt U., Marsch S. (2001). Bacterial Synthesized Cellulose—Artificial Blood Vessels for Microsurgery. Prog. Polym. Sci..

[16] Junka A., Bartoszewicz M., Dziadas M., Szymczyk P., Dydak K., Żywicka A., Owczarek A., Bil-Lula I., Czajkowska J., Fijałkowski K. (2020). Application of Bacterial Cellulose Experimental Dressings Saturated with Gentamycin for Management of Bone Biofilm In Vitro and Ex Vivo. J. Biomed. Mater. Res. Part B Appl. Biomater..

[17] Swingler S., Gupta A., Heaselgrave W., Kowalczuk M., Radecka I. (2019). An Investigation into the Anti-Microbial Properties of Bacterial Cellulose Wound Dressings Loaded with Curcumin:Hydroxypropyl-β-Cyclodextrin Supramolecular Inclusion Complex. Access Microbiol..

[18] Shao W., Wang S., Liu X., Liu H., Wu J., Zhang R., Min H., Huang M. (2016). Tetracycline Hydrochloride Loaded Regenerated Cellulose Composite Membranes with Controlled Release and Efficient Antibacterial Performance. RSC Adv..

[19] de Mattos I.B., Nischwitz S.P., Tuca A.C., Groeber-Becker F., Funk M., Birngruber T., Mautner S.I., Kamolz L.P., Holzer J.C.J. (2020). Delivery of Antiseptic Solutions by a Bacterial Cellulose Wound Dressing: Uptake, Release and Antibacterial Efficacy of Octenidine and Povidone-Iodine. Burns.

[20] Fijałkowski K., Żywicka A., Drozd R., Junka A.F., Peitler D., Kordas M., Konopacki M., Szymczyk P., Rakoczy R. (2017). Increased Water Content in Bacterial Cellulose Synthesized under Rotating Magnetic Fields. Electromagn. Biol. Med..

[21] Paximada P., Dimitrakopoulou E.A., Tsouko E., Koutinas A.A., Fasseas C., Mandala I.G. (2016). Structural Modification of Bacterial Cellulose Fibrils under Ultrasonic Irradiation. Carbohydr. Polym..

[22] Wu C.N., Fuh S.C., Lin S.P., Lin Y.Y., Chen H.Y., Liu J.M., Cheng K.C. (2018). TEMPO-Oxidized Bacterial Cellulose Pellicle with Silver Nanoparticles for Wound Dressing. Biomacromolecules.

[23] Fernandes S.C.M., Sadocco P., Alonso-Varona A., Palomares T., Eceiza A., Silvestre A.J.D., Mondragon I., Freire C.S.R. (2013). Bioinspired Antimicrobial and Biocompatible Bacterial Cellulose Membranes Obtained by Surface Functionalization with Aminoalkyl Groups. ACS Appl. Mater. Interfaces.

[24] Ávila Ramírez J.A., Gómez Hoyos C., Arroyo S., Cerrutti P., Foresti M.L. (2016). Acetylation of Bacterial Cellulose Catalyzed by Citric Acid: Use of Reaction Conditions for Tailoring the Esterification Extent. Carbohydr. Polym..

[25] Lopes T.D., Riegel-Vidotti I.C., Grein A., Tischer C.A., de Sousa Faria-Tischer P.C. (2014). Bacterial Cellulose and Hyaluronic Acid Hybrid Membranes: Production and Characterization. Int. J. Biol. Macromol..

[26] Dehnad D., Mirzaei H., Emam-Djomeh Z., Jafari S.M., Dadashi S. (2014). Thermal and Antimicrobial Properties of Chitosan–Nanocellulose Films for Extending Shelf Life of Ground Meat. Carbohydr. Polym..

[27] Indriyati, Frecilla N., Nuryadin B.W., Irmawati Y., Srikandace Y. (2020). Enhanced Hydrophobicity and Elasticity of Bacterial Cellulose Films by Addition of Beeswax. Macromol. Symp..

[28] Sommer A., Staroszczyk H., Sinkiewicz I., Bruździak P. (2021). Preparation and Characterization of Films Based on Disintegrated Bacterial Cellulose and Montmorillonite. J. Polym. Environ..

[29] Ellis J.D., Graham J.R., Mortensen A. (2013). Standard Methods for Wax Moth Research. J. Apic. Res..

[30] Wen X., Zheng Y., Wu J., Yue L., Wang C., Luan J., Wu Z., Wang K. (2015). In Vitro and In Vivo Investigation of Bacterial Cellulose Dressing Containing Uniform Silver Sulfadiazine Nanoparticles for Burn Wound Healing. Prog. Nat. Sci. Mater. Int..

[31] Bryszewska M.A., Tabandeh E., Jędrasik J., Czarnecka M., Dzierżanowska J., Ludwicka K. (2023). SCOBY Cellulose Modified with Apple Powder—Biomaterial with Functional Characteristics. Int. J. Mol. Sci..

[32] Cichosz S., Masek A. (2020). IR Study on Cellulose with the Varied Moisture Contents: Insight into the Supramolecular Structure. Materials.

[33] Ciolacu D., Chiriac A.I., Pastor F.I.J., Kokol V. (2014). The Influence of Supramolecular Structure of Cellulose Allomorphs on the Interactions with Cellulose-Binding Domain, CBD3b from Paenibacillus Barcinonensis. Bioresour. Technol..

[34] Agustin S., Wahyuni E.T., Suparmo S., Supriyadi S., Cahyanto M.N. (2021). Incorporation of Pectin during Biosynthesis of Bacterial Cellulose by Gluconacetobacter Xylinus InaCC B404: Possibility for Producing Green Food Packaging. Biodiversitas J. Biol. Divers..

[35] Kumar R., Singh A., Garg N., Siril P.F. (2018). Solid Lipid Nanoparticles for the Controlled Delivery of Poorly Water Soluble Non-Steroidal Anti-Inflammatory Drugs. Ultrason. Sonochem..

[36] Kimura F., Umemura J., Takenaka T. (1986). FTIR–ATR Studies on Langmuir–Blodgett Films of Stearic Acid with 1–9 Monolayers. Langmuir.

[37] Huang C., Yang X.Y., Xiong L., Guo H.J., Luo J., Wang B., Zhang H.R., Lin X.Q., Chen X.D. (2015). Evaluating the Possibility of Using Acetone-butanol-ethanol (ABE) Fermentation Wastewater for Bacterial Cellulose Production by Gluconacetobacter Xylinus. Lett. Appl. Microbiol..

[38] Sun D., Yang J., Wang X. (2010). Bacterial Cellulose/TiO_2_ Hybrid Nanofibers Prepared by the Surface Hydrolysis Method with Molecular Precision. Nanoscale.

[39] Dayal M.S., Goswami N., Sahai A., Jain V., Mathur G., Mathur A. (2013). Effect of Media Components on Cell Growth and Bacterial Cellulose Production from Acetobacter Aceti MTCC 2623. Carbohydr. Polym..

[40] Yassine F., Bassil N., Chokr A., El Samrani A., Serghei A., Boiteux G., El Tahchi M. (2016). Two-Step Formation Mechanism of Acetobacter Cellulosic Biofilm: Synthesis of Sparse and Compact Cellulose. Cellulose.

[41] Castro C., Vesterinen A., Zuluaga R., Caro G., Filpponen I., Rojas O.J., Kortaberria G., Gañán P. (2014). In Situ Production of Nanocomposites of Poly (Vinyl Alcohol) and Cellulose Nanofibrils from Gluconacetobacter Bacteria: Effect of Chemical Crosslinking. Cellulose.

[42] Kim H., Song J.E., Kim H.R. (2021). Comparative Study on the Physical Entrapment of Soy and Mushroom Proteins on the Durability of Bacterial Cellulose Bio-leather. Cellulose.

[43] Provin A.P., dos Reis V.O., Hilesheim S.E., Bianchet R.T., de Aguiar Dutra A.R., Cubas A.L.V. (2021). Use of Bacterial Cellulose in the Textile Industry and the Wettability Challenge—A Review. Cellulose.

[44] Ahadian S., Mohseni M., Moradian S. (2009). Ranking Proposed Models for Attaining Surface Free Energy of Powders Using Contact Angle Measurements. Int. J. Adhes. Adhes..

[45] Rouhi M., Garavand F., Heydari M., Mohammadi R., Sarlak Z., Cacciotti I., Razavi S.H., Mousavi M., Parandi E. (2024). Fabrication of Novel Antimicrobial Nanocomposite Films Based on Polyvinyl Alcohol, Bacterial Cellulose Nanocrystals, and Boric Acid for Food Packaging. J. Food Meas. Charact..

[46] Desbois A.P. (2012). Potential Applications of Antimicrobial Fatty Acids in Medicine, Agriculture and Other Industries. Recent Pat. Anti Infect. Drug Discov..

[47] Kumar P., Lee J.H., Beyenal H., Lee J. (2020). Fatty Acids as Antibiofilm and Antivirulence Agents. Trends Microbiol..

[48] Vandorou M., Plakidis C., Tsompanidou I.M., Adamantidi T., Panagopoulou E.A., Tsoupras A. (2024). A Review on Apple Pomace Bioactives for Natural Functional Food and Cosmetic Products with Therapeutic Health-Promoting Properties. Int. J. Mol. Sci..

[49] Lyu F., Luiz S.F., Azeredo D.R.P., Cruz A.G., Ajlouni S., Ranadheera C.S. (2020). Apple Pomace as a Functional and Healthy Ingredient in Food Products: A Review. Processes.

[50] Bonnin E., Garnier C., Ralet M.C. (2014). Pectin-Modifying Enzymes and Pectin-Derived Materials: Applications and Impacts. Appl. Microbiol. Biotechnol..

[51] Nishiyama Y. (2018). Molecular Interactions in Nanocellulose Assembly. Philos. Trans. R. Soc. A Math. Phys. Eng. Sci..

[52] Chen P., Nishiyama Y., Mazeau K. (2014). Atomic Partial Charges and One Lennard-Jones Parameter Crucial to Model Cellulose Allomorphs. Cellulose.

[53] Loerbroks C., Rinaldi R., Thiel W. (2013). The Electronic Nature of the 1,4-β-Glycosidic Bond and Its Chemical Environment: DFT Insights into Cellulose Chemistry. Chem. A Eur. J..

[54] Cardamone S., Popelier P.L.A. (2015). Prediction of Conformationally Dependent Atomic Multipole Moments in Carbohydrates. J. Comput. Chem..

[55] Wohlert M., Benselfelt T., Wågberg L., Furó I., Berglund L.A., Wohlert J. (2022). Cellulose and the Role of Hydrogen Bonds: Not in Charge of Everything. Cellulose.

[56] Jarvis M.C. (2022). Hydrogen Bonding and Other Non-Covalent Interactions at the Surfaces of Cellulose Microfibrils. Cellulose.

[57] Igarashi T., Hoshi M., Nakamura K., Kaharu T., Murata K.I. (2020). Direct Observation of Bound Water on Cotton Surfaces by Atomic Force Microscopy and Atomic Force Microscopy-Infrared Spectroscopy. J. Phys. Chem. C.

[58] Salmén L., Stevanic J.S., Holmqvist C., Yu S. (2021). Moisture Induced Straining of the Cellulosic Microfibril. Cellulose.

[59] Żenkiewicz M. (2000). Adhezja i Modyfikowanie Warstwy Wierzchniej Tworzyw Wielkocząsteczkowych.

[60] Rudawska A., Jacniacka E. (2009). Analysis for Determining Surface Free Energy Uncertainty by the Owen-Wendt Method. Int. J. Adhes. Adhes..

[61] Chandel V., Vaidya D., Kaushal M., Gupta A., Verma A. (2016). Standardization of Eco-Friendly Technique for Extraction of Pectin from Apple Pomace. Indian J. Nat. Prod. Resour..

[62] (1997). Geometrical Product Specifications (GPS)-Surface Texture: Profile Method-Rules and Procedures for the Assessment of Surface Texture (ISO 4288:1996).

[63] R: The R Project for Statistical Computing. https://www.r-project.org/.

[64] CRAN—Package Agricolae. https://cran.r-project.org/web/packages/agricolae/index.html.

